# Latitudinal gradients in population growth do not reflect demographic responses to climate

**DOI:** 10.1002/eap.2242

**Published:** 2021-01-18

**Authors:** Megan L. DeMarche, Graham Bailes, Lauren B. Hendricks, Laurel Pfeifer‐Meister, Paul B. Reed, Scott D. Bridgham, Bart R. Johnson, Robert Shriver, Ellen Waddle, Hannah Wroton, Daniel F. Doak, Bitty A. Roy, William F. Morris

**Affiliations:** ^1^ Plant Biology Department University of Georgia Athens Georgia 30606 USA; ^2^ Institute of Ecology and Evolution University of Oregon Eugene Oregon 97403 USA; ^3^ Department of Geography University of Oregon Eugene Oregon 97403 USA; ^4^ Department of Landscape Architecture University of Oregon Eugene Oregon 97403 USA; ^5^ Department of Natural Resources and Environmental Science University of Nevada Reno Nevada 89557 USA; ^6^ Environmental Studies Program University of Colorado Boulder Boulder Colorado 80309 USA; ^7^ Ecology and Evolutionary Biology Department University of Colorado Boulder Colorado 80309 USA; ^8^ Biology Department Duke University Durham North Carolina 27710 USA

**Keywords:** *Achnatherum lemmonii*, climate change, *Danthonia californica*, demography, distribution, *Festuca roemeri*, integral projection model, latitude, population growth, space‐for‐time

## Abstract

Spatial gradients in population growth, such as across latitudinal or elevational gradients, are often assumed to primarily be driven by variation in climate, and are frequently used to infer species’ responses to climate change. Here, we use a novel demographic, mixed‐model approach to dissect the contributions of climate variables vs. other latitudinal or local site effects on spatiotemporal variation in population performance in three perennial bunchgrasses. For all three species, we find that performance of local populations decreases with warmer and drier conditions, despite latitudinal trends of decreasing population growth toward the cooler and wetter northern portion of each species’ range. Thus, latitudinal gradients in performance are not predictive of either local or species‐wide responses to climate. This pattern could be common, as many environmental drivers, such as habitat quality or species’ interactions, are likely to vary with latitude or elevation, and thus influence or oppose climate responses.

## Introduction

Policy makers and managers increasingly ask that ecologists predict how species will respond to changing climate across their ranges. Many studies correlate species’ occurrence or demography with spatial variation in climate to understand and forecast how species will respond to climate change. A key assumption of this approach is that spatial climate patterns drive variation in population growth and hence occurrence. However, many other environmental drivers also influence population growth (reviewed in Ehrlén et al. 2016), and at least some of these are likely to vary spatially. This raises an important question: how much of the spatial variation in species’ demography can be explained by differences in average climate? If the answer is most of it, then we would be justified in using spatial correlations between occurrence or demography and average climate to forecast responses to climate change. Alternatively, responses to climate over time could be quite different from those implied by spatial patterns.

**Fig. 1:**
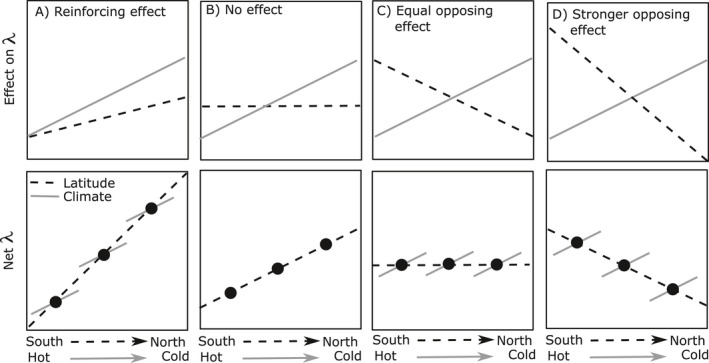
Conceptual diagram showing how latitudinal variation in environmental drivers can alter inference of climate responses. In the top row, population growth λ increases linearly with colder and wetter conditions (gray solid lines), but other environmental drivers (soils, competitors, enemies, etc.) may also vary with latitude and affect population growth in ways that reinforce or oppose climate patterns with latitude (black dashed lines). In the bottom row, the joint effects of climate and other environmental factors can alter inferred patterns of climate responses either when sampling variation across sites (black dashed lines) or when sampling variation across years within a site (gray solid lines). In the extreme, opposing climate responses within sites vs. latitudinal trends across sites can generate an example of Simpson’s Paradox (panel D), in which the direction of inference depends on the level of study (in this case, within vs. across sites).

If demography is primarily driven by climate, then we would expect to see consistent spatial gradients in population growth across species’ latitudinal and elevational ranges, corresponding to geographic patterns of average climate (Fig. 1). Indeed, this is the basis of the hypothesis that population growth will decrease in the equatorial and low‐elevation portions of a species’ range (i.e., the trailing edge) and increase in the polar and high‐elevation portions (i.e., the leading edge) as the climate warms (Parmesan et al. 1999). A corollary to this prediction, and the basis of most approaches to forecasting range shifts, is that correlations in current spatial patterns in performance with macroclimate summary statistics (e.g., Hijmans et al. 2005, PRISM Climate Group 2020) are due to *causal* effects of climate, and so can be used to infer species’ responses to climate change.

However, other non‐macroclimate factors, such as soil effects, biotic interactions, or unmeasured aspects of microclimate, may also drive spatial patterns in performance (Chardon et al. 2015, Louthan et al. 2015, Benning et al. 2019, Ford and HilleRisLambers 2019, Oldfather et al. 2019). Perhaps most importantly, non‐climate environmental drivers may also vary consistently with latitude, elevation, or aridity (Sexton et al. 2009, HilleRisLambers et al. 2013, Louthan et al. 2018, Ford and HilleRisLambers 2019), potentially limiting our ability to infer climate responses from spatial patterns (Fig. 1).

The best approach to identifying climate responses is to experimentally manipulate climate across spatial gradients, allowing one to build demographic models that directly account for effects of climate versus other forces that can influence population growth. But this approach is exceedingly difficult to implement across large spatial scales or for multiple or long‐lived species. Thus, our understanding of the spatial patterns of population growth largely stems from observational data (e.g., Menges and Dolan 1998, Doak and Morris 2010, Eckhart et al. 2011, Vanderwel et al. 2013, Diez et al. 2014, Merow et al. 2014, Oldfather and Ackerly 2018, Sheth and Angert 2018). Rather than simply regressing demographic rates against local climate using multisite and/or multiyear data, an alternative approach, that we take here, is to analyze observational data across multiple years, sites, and species spanning a latitudinal gradient using mixed models to parse the effects of climate variables, latitudinal effects not explained by climate, and other random site effects not captured by latitude. While this approach does not allow us to dissect which non‐climatic drivers might contribute to latitudinal or local site effects, it does allow a measure of how much spatial variation in population growth is due to macroclimate vs. other factors that may or may not vary with latitude. By taking a demographic approach, we are also able to integrate the effects of different drivers on multiple demographic rates into an overall effect on population growth (Doak and Morris 2010, Louthan et al. 2018, Oldfather and Ackerly 2018).

This approach is also valuable for testing the generality of environmental drivers of population growth. In the current era of unprecedented global change, ecologists increasingly need to make generalizable predictions of how species will respond. Many studies organize species by key traits (Suding et al. 2008, Reu et al. 2011), functional groups (Elmendorf et al. 2012, Boulangeat et al. 2014), or habitat types (Hamann and Wang 2006, Hamann and Aitken 2013) to predict general responses to environmental change. However, other studies have emphasized the idiosyncratic nature of species‐level responses (Adler et al. 2006, Dalgleish et al. 2011, Rapacciuolo et al. 2014, Shriver 2016). Comparative demographic studies are necessary to test the extent to which species in the same habitats or functional groups share common demographic drivers, and thus may respond similarly to environmental change.

Here we quantify and compare the spatiotemporal patterns and drivers of population growth of three C_3_ perennial bunchgrasses native to upland prairie habitats in the Pacific Northwest (PNW), USA, a region characterized by mild winters and warm, dry summers. Prairies are an endangered ecosystem in this region (Noss et al. 1995), historically dominated by perennial bunchgrasses and forbs (Christy and Alverson 2011). Throughout the PNW, prairies have been substantially impacted by land‐use changes and invasive species, and prairie species may be particularly susceptible to any further stresses imposed by climate change. Yet, the extent to which prairie species share similar demographic drivers is unknown, as is the importance of climate vs non‐climate effects on population growth. We used detailed demographic data collected across four annual censuses (three transitions) at multiple sites spanning a 684‐km latitudinal gradient to address the following questions:

1. What are the spatiotemporal patterns of demographic and population growth rates and are they similar across species? Are spatial patterns in performance consistent with leading‐trailing edge predictions?
2. Do macroclimate variables generate most or all of any latitudinal variation in performance, or do other unmeasured effects drive important variation in performance? If climate effects are detected, do they explain or oppose any spatial gradients in performance?

## Methods

### Study species and sites

**Fig. 2:**
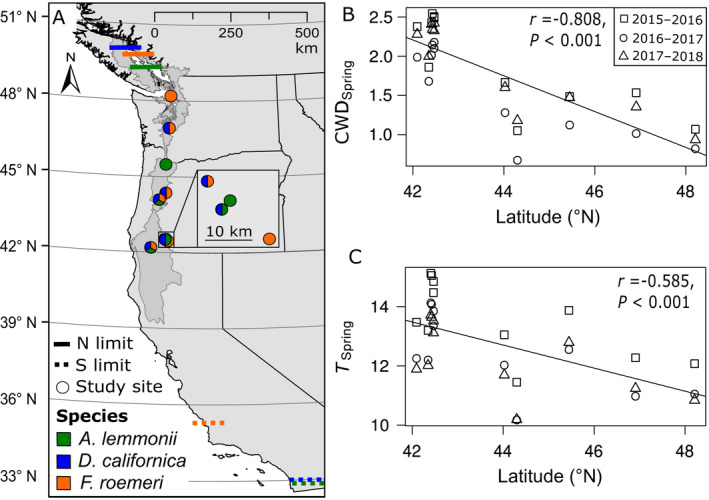
(A) Map of the study sites used for demographic monitoring of three perennial bunchgrasses in Oregon and Washington, USA (see also Appendix S1: Table S1). Several sites contained study populations for more than one species (divided circles). In total, we monitored five, six, and seven populations, respectively, of *Achnatherum lemmonii*, *Danthonia californica*, and *Festuca roemeri* from 2015 to 2018 along a latitudinal gradient. Lines show the estimated latitudinal range limits for each species in the study area (i.e., prairies west of the Cascade and Sierra mountain divides [darker gray shading shows the extent of primary ecoregions in which prairies are located], see Appendix S1 for details). Note that the northern range limit for *Achnatherum* is driven by scattered occurrences in coastal British Columbia, but this species is exceedingly rare north of the Willamette Valley in Oregon (~45.5° N). Across all study populations and years, (B) spring climatic water deficit (CWD_Spring_) and (C) mean temperature (*T*_Spring_) decrease with latitude. Conditions tended to be warmer during the 2015–2016 annual transition, and cooler and wetter in the 2016–2017 transition, compared to other years in the study. Other climate variables (see *Methods*) were not significantly correlated with latitude (Appendix S1: Fig. S2s

We studied three native perennial bunchgrasses: *Achnatherum lemmonii* (Vasey) Barkworth var. *lemmonii*, *Danthonia californica* Bol., and *Festuca roemeri* Y.V. Alexeev. All three species were historically widespread in prairie grasslands in the PNW (Christy and Alverson 2011). We monitored demographic performance in 5–7 populations for each species (across 10 sites total) along a latitudinal gradient extending from roughly the latitudinal center of each species’ range toward its northern range limit (Fig. 2, Appendix S1: Table S1).

### Demographic data collection

In 2015, we established permanent transects in each population, which we used to map and tag all individuals of a given species (*N* = 207–284 per population). All individuals were censused each year between 1 May and 24 June from 2015 to 2018; censuses were timed to coincide with seed development so that inflorescence counts were reflective of annual reproductive output. During each census, we recorded the survival, size, and inflorescence production of all individuals and searched for new seedlings in recruitment plots (see Appendix S1).

### Analysis of vital rates

#### Overview

We took two approaches to estimate size‐dependent vital rates. First, we used generalized linear models with fixed site and year effects (hereafter, “categorical models”) to most flexibly fit the best‐supported vital rate functions for each annual transition (e.g., by allowing size × site × year interactions and avoiding distributional assumptions of site effects). Second, we fit mixed models to parse the fixed effects of climate and latitude as well as random effects of site in explaining variation in vital rates (hereafter, “climate models”). We outline these approaches in more detail in the following section.

#### Vital rate models

We modeled the probabilities of survival, reproduction (i.e., producing at least one inflorescence), and seedling survival with logistic regression and, for reproductive plants, the number of inflorescences using a negative binomial regression. All analyses were performed in R v. 3.6.0 (R Core Team, 2017).

We modeled growth as the distribution of size in year *t* + 1 as a function of size in year *t*. Although growth is commonly modeled with a normal distribution, size transitions in our data did not meet the assumption of normality after transformation (Appendix S1: Fig. S1). Consequently, we used two approaches to account for skewed growth. First, we modeled the size distribution of surviving seedlings using the empirical probability density. Second, we modeled the size transitions of all other individuals using an approach that combines quantile and beta regressions (see Appendix S1; DeMarche et al. 2019).

We estimated the number of new seedlings per inflorescence the prior year using negative binomial models. For each 25 × 25 cm recruitment plot, we summed the total number of inflorescences produced within the surrounding 50 × 50 cm plot the prior year and divided this value by four to get an average inflorescence density in the recruitment plot. We modeled recruitment using the number of seedlings as our response variable and including the average inflorescence density as a covariate (e.g., anonymous reproduction; Caswell 2001). This approach produced more realistic recruitment rates than assuming a constant number of recruits per inflorescence (see Appendix S1).

#### Analysis of year‐ and site‐specific vital rates

We first fit categorical vital rate models with fixed effects of site and year and, for size‐dependent rates, both linear and quadratic effects of size. We considered models with all two‐ and three‐way interactions and compared nested models with the Akaike information criterion corrected for sample size (AIC_c_) using the MuMIn package (Barton 2014) to identify the best‐supported model for each vital rate (Burnham and Anderson 2004). In some cases, quadratic size effects resulted in biologically unrealistic patterns at the tails of the size range where data were sparse and were removed from the models. Sample sizes for estimating seedling survival rates were low in many sites and years, so we only considered effects of site for *Achnatherum* and *Danthonia*, and estimated a single species‐wide seedling survival rate for *Festuca*. Given the low number of surviving seedlings, we modeled a single recruit size distribution for each species.

#### Climate and spatial effects on demographic performance

We next tested for climate drivers of demographic performance across sites and years for each species. We focused on climate variables related to temperature, precipitation, and drought stress because of their demonstrated effects on PNW prairie plant performance (Pfeifer‐Meister et al. 2016, Reed et al. 2019). We calculated five seasonal climate variables using data from PRISM (PRISM Climate Group 2020) and SSURGO USDA: mean temperature (*T*), total precipitation (*P*), actual evapotranspiration (AET), climatic water deficit (CWD), and the first principal component of temperature and precipitation (PC1; increasing values indicate warmer/drier conditions, explains 84%, 88%, and 76% of the variance for *Achnatherum*, *Danthonia*, and *Festuca*, respectively). AET reflects the amount of water removed by evaporation and transpiration, CWD is the deficit of water that could potentially be removed vs. actually removed, and PC1 captures the variance in temperature and precipitation across sites and years. We aggregated these climate variables into seasonal values for winter (November–February) and spring (March–June; see Appendix S1). These species germinate in the winter, grow and reproduce in the spring, and are largely senescent during the dry summer months.

We tested for climate effects on vital rates by fitting a series of mixed models that substituted climate variables for fixed site and year effects, while accounting for other potential environmental drivers with a fixed effect of latitude and a random site effect. The latitude effect captures any non‐macroclimate environmental drivers that are correlated with latitude, whereas the random site effect captures other unexplained local site effects while allowing the model to also estimate latitudinal trends. Although spatial effects could also be modeled with only a random site effect, preliminary analyses showed that these models yielded random site coefficients that were strongly correlated with latitude for some vital rates (Appendix S1: Fig. S3), so we chose to also include latitude as an explanatory variable. We included site as a random effect in these models because of our primary interest in estimating climate and latitude effects; however, models that included site as a fixed effect yielded very similar results (Appendix S1: Table S2).

For each vital rate, we fit models that included linear and quadratic effects of climate, linear, and quadratic effects of size, latitude, and all two‐way interactions. Quadratic climate effects were included to allow unimodal relationships between vital rates and climate. Given the small number of sites and years with which to identify climate drivers, we were unable to include multiple climate variables within a single model; however, CWD, AET, and PC1 incorporate effects of both temperature and precipitation in different ways. We considered the effects of spring climate variables on all vital rates, and, because germination occurs earlier in the year, we also considered the effects of winter climate variables (in the appropriate year) on recruitment and seedling survival. Latitude and climate variables were often correlated (Appendix S1: Fig. S2); such collinearity among predictor variables can inflate the standard errors of regression coefficients. However, simply removing collinear variables is not recommended, as it can alter the estimated effects of the retained variables (Freckleton 2010), and would also prevent us from addressing our core questions. For these reasons, we retained both climate and latitude variables in our regressions, but note that the coefficients and standard errors should be interpreted cautiously. We fit GLMMs using the lme4 package (Bates et al. 2015) and used AIC_c_ to identify the best‐supported model for each vital rate. We are not aware of methods to incorporate random effects for both the mean and precision parameters of a beta regression, so we included site as a fixed effect influencing both parameters in our growth models and used the average of the site coefficients to generate species‐level predictions of climate effects.

### Population models

We constructed size‐structured population models in two ways: (1) using the best‐supported categorical vital rate models to build population models for each annual transition and site, and (2) using the best‐supported climate vital rate mixed models to construct population models as functions of environmental drivers.

We modeled the fates of plants older than seedlings using 200 evenly divided size classes spanning the minimum and maximum sizes observed for each species (± 0.001) and included a separate class for seedlings. We parameterized models by discretizing vital rate functions using the midpoint size for each class (e.g., IPMs; Easterling et al. 2000, Ellner and Rees 2006), and estimated growth probabilities of adults and recruits as the differences of the cumulative density function at class boundaries (as in Dibner et al. 2019). We estimated the elasticities of population growth to the underlying vital rates by perturbing their values (see Appendix S1).

### Spatiotemporal patterns in demography

We used population models constructed from the categorical vital rate models with fixed site and year effects described above to investigate each species’ spatiotemporal patterns of population growth. We calculated annual population growth rates (λ) as the dominant eigenvalue of each discretized IPM matrix and the transient stochastic population growth rate (λ_s_) for each site by starting at the stable stage distribution of the mean matrix for that site and randomly sampling from the three annual matrices for 100 time steps. To account for parameter uncertainty, we estimated 95% bias‐corrected confidence intervals by resampling the coefficients of each vital rate function using their covariance matrices 1000 times and re‐estimating λ and λ_s_. We explored spatial patterns in performance by fitting ANCOVAs for λ or λ_s_ with latitude, species, and their interaction as explanatory variables.

### Effects of climate

We explored how population growth varies as a function of both climate and latitude effects. We sampled 2,000 sets of correlated climate values from a multivariate normal distribution defined by the means and covariances of climate variables in the data set. These multivariate climate conditions were used as drivers to obtain estimates of λ across the observed range of latitudes for each species. We also tested the contribution of specific vital rates to changes in λ with latitude or climate by varying each vital rate separately while holding all other vital rates constant at their mean value. Finally, we compared the ability of climate and latitude effects to explain the observed variation in population growth among sites and years. For mixed models, we compared the variance explained by the fixed effects (e.g., climate and latitude) vs. the variance explained by both the fixed effects and the random site effect (i.e., conditional vs. marginal *R* ^2^, respectively; Nakagawa and Schielzeth 2013). We also compared the variance explained by the best‐supported climate and latitude models (with random site effects) to that explained by categorical models that allow for site × year interactions.

## Results

### Spatiotemporal patterns in demography

**Fig. 3:**
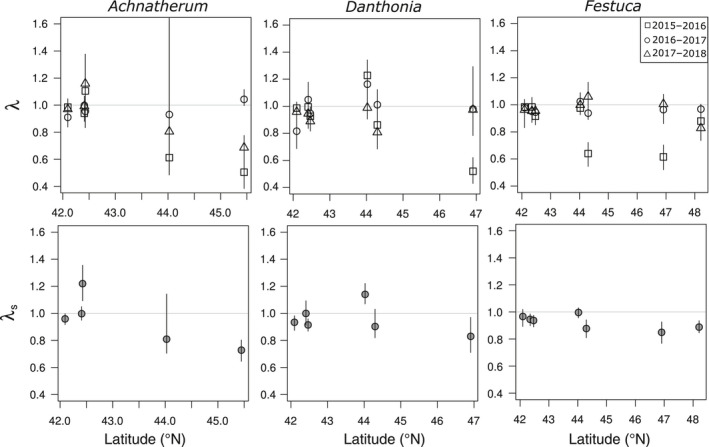
Spatiotemporal patterns of population growth for three perennial bunchgrasses. The top row shows annual population growth rates from 2015 to 2018 along a latitudinal gradient, with 95% bias‐corrected confidence intervals from 1,000 bootstrap replicates including parameter uncertainty. The bottom row shows mean and 95% confidence intervals for transient stochastic population growth rates λ_s_ at each site from random sampling of each annual matrix for 100 time steps and 1,000 replicates including parameter uncertainty.

Patterns of population growth were highly variable across both sites and years in all three species (Fig. 3). We observed catastrophic years, with an annual population growth rate (λ) of <0.7, in at least one site for every species. These low growth rates largely occurred during the 2015–2016 transition, which was warmer and drier than other years in the study (Fig. 2). *Danthonia* had the greatest variability in population growth, with λ ranging from 0.52–1.23, followed closely by *Achnatherum* (λ 0.50–1.16). *Festuca* was also prone to catastrophic years, but we did not observe any boom years for this species (λ 0.62–1.06). We only observed extremely low λ estimates at more northern sites, whereas λ estimates at the lowest latitudes tended to be clustered around 1; these trends were also reflected in lower stochastic population growth rates at higher latitudes (Fig. 3). Overall, latitude had a significantly negative effect on population growth rates (λ *F*_1,48_ = 10.23, *P* = 0.002; λ_s_ *F*_1,12_ = 10.12, *P* = 0.008; Appendix S1: Table S3). This trend was consistent among species (slopes = −0.107 to −0.015), although the interaction between latitude and species was marginally significant (λ *F*_2,48_ = 2.85, *P* = 0.068; λ_s_: *F*_2,12_ = 3.10, *P* = 0.082).

### Climate drivers of demographic performance

We detected strong effects of climate on vital rates for all three species (Table 1, Appendix S1: Table S4). However, climate alone did not explain variation in several key vital rates, for which we found strong support for latitude effects in addition to climate (Table 1). Overall, the best‐supported climate vital rate models generally had high conditional *R* ^2^ values (i.e., the variance explained by both fixed and random effects) relative to the *R* ^2^ values of the categorical models; although these different *R* ^2^ metrics are not quantitatively comparable, their relative values suggest that the climate vital rate models explained a substantial fraction of the variance captured by categorical models (Appendix S1: Table S2). Most of this variance was explained by fixed effects (c.f. marginal *R* ^2^ and conditional *R* ^2^, Appendix S1: Table S2), suggesting that climate and latitude variables together were able to capture much of the spatiotemporal variation in performance. However, random site effects, representing local or unexplained environmental drivers, explained a substantial fraction of the variance in inflorescence production for *Festuca* and *Achnatherum*, recruitment for *Festuca*, and seedling survival for *Achnatherum* and *Danthonia*.

**Table 1:** The best‐supported climate‐based vital rate models with or without considering a fixed effect of latitude for three perennial bunchgrasses from a series of mixed models using size, latitude (Lat), and climate variables as fixed effects and treating site as a random effect.

Vital rate	*N*	Best‐supported	LL	Without latitude	LL	Δ
*Achnatherum lemmonii*						
Survival of established plants	3086	Size × (PC1_Spring_ + PC1_Spring_^2^) + Lat × PC1_Spring_	−1039.2	Size × CWD_Spring_ + CWD_Spring_^2^	−1067.8	51.3
Growth	2408	μ: Size + CWD_Spring_ + CWD_Spring_^2^	898.1			
‐	‐	ϕ: Size + CWDSpring + CWDSpring2				
Reproduction	4357	CWD_Spring_ × (Size + Size^2^)	−1395.1			
Inflorescences	2368	PC1_Spring_ × (Size + Lat) + Size^2^ + Lat × PC1_Spring_^2^	−6139.9	Size × *T*_Spring_ + Size^2^	−6152.1	16.3
Recruitment	320	*T*_Winter_	−496.0			
Seedling survival	274	*T*_Winter_	−128.3			
*Danthonia californica*						
Survival of established plants	3007	CWD_Spring_ × (Size + Lat)	−886.8	Size × CWD_Spring_ + CWD_Spring_^2^	−894.0	12.4
Growth	2575	μ: Size × CWD_Spring_ + CWD_Spring_^2^	1084.9			
‐	‐	ϕ: Size + CWDSpring				
Reproduction	4021	PC1_Spring_ × (Size^2^ + Lat) + PC1_Spring_^2^ × (Size + Lat)	−1759.9	PC1_Spring_ × (Size^2^) + PC1_Spring_^2^ × Size	−1781.9	38.0
Inflorescences	2239	Size + Size^2^ × (*P*_Spring_ + *P*_Spring_^2^)	−5623.0			
Recruitment	332	*P*_Spring_	−496.0			
Seedling survival	330	*P*_Spring_	−174.4			
*Festuca roemeri*						
Survival of established plants	3985	CWD_Spring_ × (Size + Lat)	−832.2	Size × CWD_Spring_ + CWD_Spring_^2^	−840.4	14.4
Growth	3563	μ: Size × (*T*_Spring_ + *T*_Spring_^2^),	1925.8			
		ϕ: Size + TSpring				
Reproduction	5494	Size + Size^2^ + Lat × (PC1_Spring_ + PC1_Spring_^2^)	−2708.0	Size + Size^2^ + PC1_Spring_ + PC1_Spring_^2^	−2715.2	8.5
Inflorescences	2264	Lat × CWD_Spring_ + Size^2^	−5970.1	Size + Size^2^ + CWD_Spring_	−5989.6	35.0
Recruitment	530	*T*_Spring_	−461.3			
Seedling survival	96	AET_Spring_	−48.7			

Notes

If the best‐supported model included latitude, then the next‐best model without latitude is given for comparison on the right‐hand side. *N* gives the sample size for each species and vital rate used in model‐fitting. LL gives the log‐likelihood of each model and Δ gives the difference in AIC_c_ (Akaike information criterion corrected for sample size) relative to the best‐supported model. Note: climate‐based growth models always include site as a fixed effect, rather than a random effect, for both the mean (μ) and precision (ϕ). Recruitment models always include inflorescence density as a predictor. The × indicates an interaction between two variables, such that the main effects are also included in the model. Parentheses indicate interactions with all variables inside the parentheses.

**Fig. 4:**
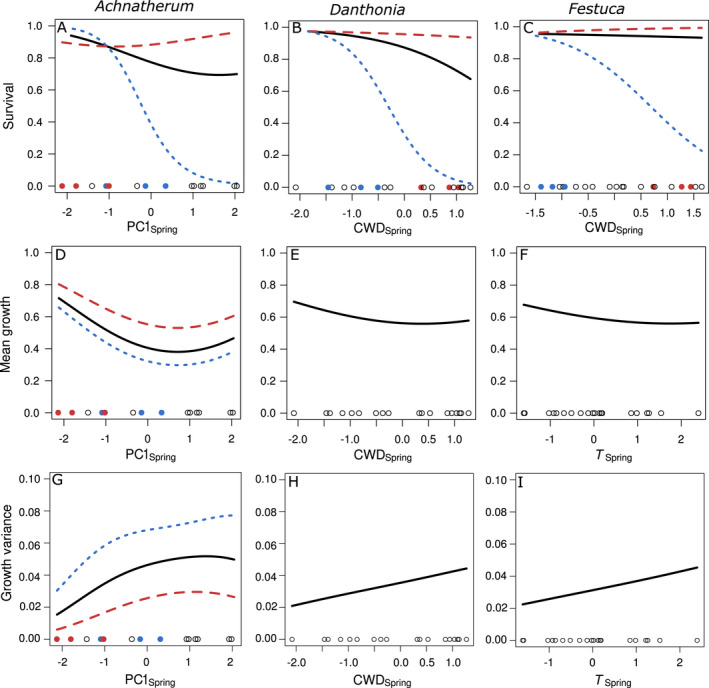
Fitted survival and growth responses to climate drivers across three perennial bunchgrasses (A‐I). Lines give the predicted vital rate responses from the best‐supported climate models (Table 1), and points show the distribution of climate values observed across sites and years. Vital rates are shown for average‐sized individuals and the mean latitude across study sites for each species (black solid lines). Where there were significant latitude by climate interactions, we also show vital rates for the minimum (red dashed) and maximum (blue dotted) latitudes across study sites for each species, and highlight the observed climate values for these sites (red, minimum, blue, maximum). For *Achnatherum*, site intercepts for growth mean and variance were significantly correlated with latitude (see *Results*), so we also show growth rates predicted for the lowest latitude (red dashed) and highest latitude (blue dotted) sites. Climate variables are standardized to mean 0 and variance 1. Note that growth mean and variance are shown on a transformed scale as a proportion of the range of possible sizes based on a species‐ and size‐specific minimum and maximum value (see Appendix S1). Survival and growth are shown here because they have the greatest effects on population growth rates; see Appendix S1: Fig. S4 for additional vital rate climate relationships.

Climate variables related to drought stress were most commonly selected as the strongest predictors of vital rates for all three species (Table 1). Either CWD or PC1 were included in many of the best‐supported models for each species. For example, survival of established plants decreased with increasing drought stress in all three species (Fig. 4). Similarly, drought stress decreased inflorescence production in *Achnatherum* and *Festuca* and decreased growth in *Achnatherum* and *Danthonia* (Fig. 4, Appendix S1: Fig. S4). Conversely, a few vital rates increased under drought stress, such as the probability of reproduction in *Achnatherum* and *Danthonia* (Appendix S1: Fig. S4).

Other climate variables, such as temperature and precipitation, predominantly influenced rates related to reproduction and recruitment. In *Achnatherum*, seedling recruitment and subsequent survival both decreased with warmer winters (Appendix S1: Fig. S4). In *Festuca*, seedling recruitment decreased and growth decreased on average and became more variable with warmer springs (Fig. 4). *Danthonia*, by contrast, was more influenced by precipitation than temperature. Greater spring precipitation increased inflorescence production and seedling survival, but decreased recruitment.

There was also strong support for latitude in many vital rate models (Table 1). For all three species, the effect of climate on post‐seedling survival was dependent on latitude, with lower sensitivity to climate variation between years at lower latitudes (Fig. 4 top). We also observed climate x latitude interactions for the probability of reproduction in *Danthonia* and *Festuca* and for inflorescence production for *Achnatherum* and *Festuca*; these interactions also indicated reduced sensitivity to climate at lower latitudes rather than changes in the direction of climate effects (Appendix S1: Fig. S4). Although we did not find support for latitude effects on growth for any species based on AIC_c_, the coefficients for site in the best‐supported growth models for *Achnatherum* were strongly correlated with latitude for both the mean (*r* = −0.920, *P* = 0.027) and the precision (*r* = −0.935, *P* = 0.020), indicating higher and less variable growth at lower latitudes (Appendix S1: Table S5). Together, these latitudinal effects generally oppose the direction of main climate effects (Fig. 4); climate becomes more favorable (cooler and wetter) as latitude increases (Fig. 2B,C).

**Fig. 5:**
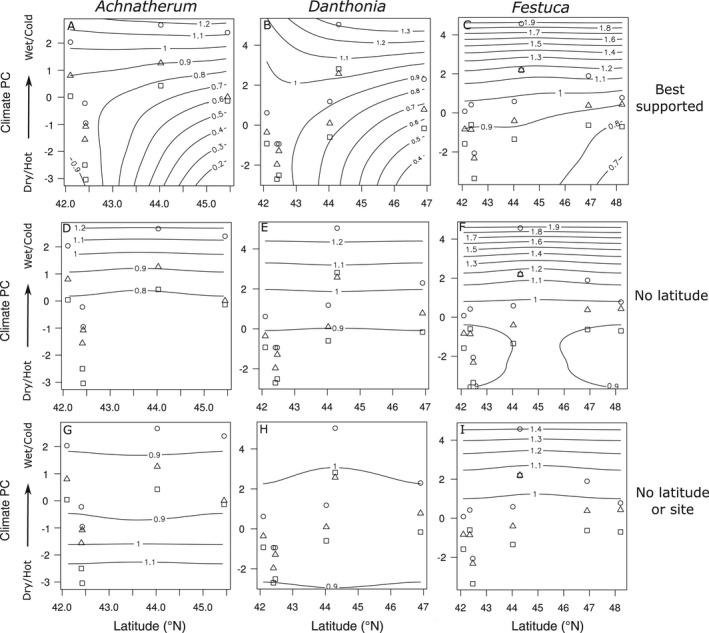
Contour plots of population growth rate as a function of climate and latitude for three perennial bunchgrasses. Climate here is shown as the first principal component of all climate variables, reflecting variation from warmer/drier to cooler/wetter conditions. Contour lines show changes in population growth rate in 0.1 increments. Predictions are from the best‐supported models allowing interactions between latitude and climate as well as a random site effect (top, A–C), the best‐supported models that exclude latitude but include a random site effect (center, D–F), and the best‐supported models that include climate but exclude latitude or site effects (bottom, G–I). Points show the distribution of latitude and climate conditions across sites and annual transitions (squares, 2015–2016; circles, 2016–2017; triangles, 2017–2018). Note that contour lines may be slightly curved even in the absence of a latitude effect due to sampling variation and the effects of aggregating different climate variables into a common PC variable on the *y*‐axis.

When integrating these climate‐based vital rate models into structured population models, the resulting population growth rates were well correlated with estimates based on categorical site x year vital rate models (Appendix S1: Table S6, Fig. S5A), suggesting that the combined climate and latitude effects capture much of the spatiotemporal variation in population growth, just as they did for underlying vital rates. Exploring the sensitivity of population growth to climate and latitude uncovered remarkable similarities across all three species. Despite differing vital rate models, the population growth of all three species decreased with latitude as well as with warmer and drier climate conditions (Fig. 5A–C). These opposing latitudinal and climate effects on population growth suggest that the climate effects uncovered here predominantly explain responses to annual climate variation within a site rather than spatial patterns across a latitudinal gradient. Within sites, population growth decreased in warmer and drier years, but among sites population growth was higher and more stable in the warmer and drier portion of the range. Further, climate effects are not constant across the range but instead interact with latitude; populations in the northern portion of the range are much more sensitive to warmer and drier conditions, driving greater variability in performance there. For all three species, these effects were largely driven by changes in survival and growth (Appendix S1: Fig. S6). Although other vital rates sometimes showed compensatory responses to climate or latitude (Appendix S1: Fig. S4), population growth was much more sensitive to survival and mean growth than to other vital rates (Appendix S1: Fig. S7), and these compensatory responses had little effect in buffering the overall effects on population growth.

Models that only considered climate drivers of performance and ignored latitude or site effects greatly underestimated and in some cases totally mischaracterized the effects of climate. For example, models that ignore all spatial effects conflate spatial gradients in performance with temporal climate responses, resulting in predicted climate effects that are extremely weak (Fig. 5G‐I) and, for *Achnatherum*, in the wrong direction (Fig. 5G). Predictions from these models do a much poorer job of capturing observed patterns of population growth, as estimated by categorical models (Appendix S1: Table S6, Fig. S5C). Models that include random site effects but not a latitude effect are better able to capture the direction of climate responses than models that only consider climate (cf. Fig. 5D, G), but do not allow for climate effects to vary with latitude and thus also fail to predict the increased sensitivity to climate seen at higher latitudes (Appendix S1: Table S6, Fig. S5B).

## Discussion

Despite identifying multiple climate variables with strong effects on vital rates, climate variation alone did not explain the spatial gradients in population growth for any of the three species in this study. Instead, we found support for strong latitudinal effects that largely oppose the spatial pattern of predicted climate effects. This result suggests that the climate effects uncovered here largely explain annual variation in performance within sites, with catastrophic years reflecting years that were particularly hot and dry for a given site, but that these climate effects were not predictive of larger spatial patterns in demographic performance, in which population growth was lowest in cooler and wetter northern sites. This result contradicts the frequent assumption that space can be substituted for climate, an assumption that is commonly made in many studies of climate responses. If we had not considered spatial effects or only measured population growth over a single annual transition, we could have erroneously interpreted the latitudinal gradient in performance as evidence that warmer and drier conditions would be beneficial for local populations (Fig. 1D). Other studies have also demonstrated that spatial relationships with climate do a poor job of predicting responses to climate change over time (Adler and Levine 2007, Rapacciuolo et al. 2012, Elmendorf et al. 2015). Although some studies have considered spatial variables such as latitude or elevation when testing for climate drivers of performance (Wang et al. 2010, Schwalm et al. 2016), this approach remains rare. Our results emphasize that the assumption that spatial gradients in performance are due to gradients in climate, and therefore predictive of responses to climate change through time, needs to be evaluated and justified in each case.

One potential explanation for the opposing climatic and latitudinal effects is that one or more important environmental drivers are correlated with latitude, modifies climate responses, and remains uncaptured in our models. We examined several potential environmental drivers for which we had data in this study, but none clearly explained the observed pattern. For example, latitude is not related to soil fertility, land management, or the presence of invasive species (Appendix S1: Table S1, Fig. S9). We did find a moderate positive correlation between community‐wide spring ground‐level NDVI (a measure of green vegetation) and latitude across our study sites (Appendix S1: Fig. S9; *r* = 0.42, *P* < 0.01); although this measure is partially driven by phenology differences (Reed et al. 2019), it could also suggest that competition might increase with latitude and perhaps contribute to decreases in performance. However, this relationship was weak and incomplete records of NDVI at our study sites prevented us from including this driver in vital rate models. Other studies in PNW prairies also point to potential explanations. For example, in a related study of fungal endophytes, pathogen loads in *Festuca* were found to increase with latitude across the same study sites; however, no latitudinal effect was found for *Danthonia* (G. Bailes, *unpublished data*). Dormancy decreases over the same latitudinal gradient for *Festuca*, but not for *Danthonia* (Roy et al., *unpublished manuscript*). Soil clay content has also been shown to decrease with latitude in our study region (Hendricks 2016). Disentangling the potential effects of competition, pathogens, and soils from those of climate would require additional experiments. Indeed, one limitation of the observational approach we use here is the difficulty in determining which other environmental factors may be driving latitudinal or local site effects on performance. In that vein, we note that a related warming experiment across the same latitudinal gradient uncovered similar climate responses as this study in *Festuca*, which showed reduced performance under warming (Reed et al., 2020 *unpublished manuscript*).

Alternatively, differing climate responses across a latitudinal gradient can be suggestive of local adaptation (Gorton et al. 2019). We observed stronger decreases in performance with warmer and drier conditions in the cooler and wetter northern sites, whereas the warmer and drier sites in the central portion of the range tended to be less sensitive to climate variation across years (Fig. 5). This could be explained by differing climate tolerances in different portions of the species’ range, with recent warm and dry years in the north possibly representing more severe departures from historical conditions. In fact, there is a trend for the driest years during the study period to be more extreme relative to historical conditions in sites at higher latitudes, though the pattern is more variable for temperature (Appendix S1: Fig. S10). Many studies have demonstrated local adaptation to climate in plants across latitudinal gradients (e.g., Joshi et al. 2001, Ågren and Schemske 2012, DeMarche et al. 2018), and this is more likely to be the rule rather than the exception. However, while observational studies such as this can identify patterns suggestive of local adaptation, such as differing climate responses or optima, transplant or common garden experiments are necessary to rigorously test for genetic adaptation in climate tolerances among populations.

Contrary to our expectation, latitudinal patterns in population growth were not consistent with leading‐trailing dynamics (e.g., Parmesan et al. 1999), which would predict decreased vulnerability to warming toward the northern range limit. Rather, we saw the opposite pattern, with more northern “leading edge” populations showing reduced performance and greater sensitivity to climate. Catastrophic years with asymptotic annual growth rates less than 0.7 were only observed at higher latitudes, and such years resulted in decreased estimates of the transient stochastic population growth rate. This pattern is instead more consistent with classic range limit theory, which predicts reduced and more variable performance toward range limits (Brown 1984, Gaston 1990, Kirkpatrick and Barton 1997). Interestingly, several meta‐analyses have failed to find general empirical support for this prediction (Sexton et al. 2009, Abeli et al. 2014, Pironon et al. 2017). Thus, although there are several studies that have also found this pattern (Levin and Clay 1984, Purves 2009, Eckhart et al. 2011, Baer and Maron 2019), it appears to be relatively rare.

An outstanding question in ecology is the extent to which species that share a habitat type or functional group can be usefully combined to predict common responses to environmental change. Although this is a much larger question than our study can fully address, it is notable that we found remarkably similar demographic responses across these three prairie bunchgrasses. All showed the greatest elasticity of population growth to survival and growth of medium to large‐sized plants, consistent with their moderate lifespan (estimated 11–26 yr; Appendix S1), and these key vital rates, and thus overall population growth, were similarly decreased under warmer and drier conditions and at higher latitudes. However, we also saw some differences among species; *Festuca* was less responsive to environmental drivers and had less variable population growth overall. Meta‐analyses across many plant species have generally found very little effect of phylogenetic, spatial, or habitat similarity between species in explaining variation in demographic performance (Buckley et al. 2010, Burns et al. 2010, Coutts et al. 2016), although Adler et al. (2014) found that key functional traits, such as seed mass and leaf economics, were predictive of overall life history strategies as measured by elasticity patterns.

Here we demonstrate an approach to test whether local climate responses explain spatial gradients in performance, using three common perennial bunchgrasses in PNW prairies. The mixed model approach that we outline is most useful for observational data sets, which are commonly used to identify climate drivers of performance, and also requires some temporal resampling within sites to disentangle spatial and climatic effects. By dissecting the effects of climate from other potential spatial gradients and local site effects, we find evidence that population growth is likely to decline with warmer and drier conditions, despite a latitudinal trend of decreasing population growth toward the cooler and wetter northern portion of each species’ range. Such a pattern (an example of Simpson's Paradox; Simpson 1951), in which spatial gradients are not indicative of local responses to climate variation over time, may be common if species are locally adapted to climate or other factors, such as species’ interactions or habitat quality, also vary spatially and mediate climate responses. For these reasons, we suggest that researchers critically evaluate the “space‐for‐time” assumption when predicting environmental drivers of performance, and that the analysis framework we present can be a useful way to do so.

## Supporting information

Appendix S1

## Data Availability

Data are available from the Dryad Digital Repository (DeMarche et al. 2020): https://doi.org/10.5061/dryad.2rbnzs7m0.
