# Supplementary material for: Latitudinal gradients in population growth do not reflect demographic responses to climate
Source: Ecol Appl. 2021 Jan 18;31(2):e2242. doi: 10.1002/eap.2242 (PMC7988552; doi:10.1002/eap.2242)
Supplement: Supplementary file 1 — Appendix S1 [file EAP-31-e2242-s001.pdf]

Supporting Information. Peterson, M.L., G. Bailes, L.B. Hendricks, L. Pfeifer-Meister, P.B. Reed, S.D. Bridgham, B.R. Johnson, R. Shriver, E. Waddle, H. Wroton, D.F. Doak, B.A. Roy, and W.F. Morris. 2020. Latitudinal gradients in population growth do not reflect demographic responses to climate. Ecological Applications.

Appendix S1: Details of demographic data and modeling

SUPPLEMENTAL METHODS

Determination of species' latitudinal range limits – We assigned species' latitudinal range limits based on recent recorded observations gathered from the Consortium of Pacific Northwest Herbaria (www.pnwherbaria.org/) and the Consortium of California Herbaria (https://ucjeps.berkeley.edu/consortium/) as of April 1, 2019. We defined species' current range limits as the latitudes of the northernmost and southernmost known populations documented within the last 50 years. Because our focal study ecosystems are westside lowland Pacific Northwest prairies, we restricted records to the west side of the Cascade and Sierra mountain divides to define the latitudinal range limits. Although *Achnatherum* is rare north of the Willamette Valley in Oregon (~ 45.5° N), it does occur in disjunct populations in the Gulf Islands and Vancouver Island of British Columbia, extending its northern range limit to approximately 49.3° N.

Demographic data collection - In 2015, we established permanent transects in each population, which we used to map and tag (with colored nails or golf tees) all individuals of a given species. The length and width of transects varied among sites depending on plant density, but included more than 200 adult plants in each population (N = 207 – 284). All individuals were censused each year between May 1 – June 24 from 2015 to 2018. During each census, we relocated all individuals to record their survival and measured the size of each individual as the

number of tillers (if < 10), or the basal area of the plant (if ≥ 10 tillers). Plant basal area was calculated using the major and minor axes of an ellipse. Large bunchgrasses often die back in the center, such that the living tissue is better described as a ring. For this reason, we measured the total area and the area of central die-back as ellipses based on perpendicular measurements of the diameters, and used the difference in these values as the area of living tissue. We also counted the number of inflorescences per plant, and searched for new seedlings in 25 x 25 cm recruitment plots distributed along each transect (N = 14 – 53 per population). New seedlings were also mapped and marked to track subsequent survival and growth. We estimated size-dependent vital rates using the log of plant area as the measure of plant size. For small plants, we translated measurements of tiller number into estimates of plant area by estimating the average area per tiller for each species from a subset of small plants with both measurements (N = 144-273, r > 0.63).

Modeling recruitment as a function of inflorescence density - We estimated the number of new seedlings per inflorescence the prior year using negative binomial models. For each 25 x 25 cm recruitment plot, we summed the total number of inflorescences produced within the surrounding 50 x 50 cm plot the prior year and divided this value by four to get an average inflorescence density in the recruitment plot. Although seed dispersal in these species is likely to occur beyond the surrounding 50 x 50 cm plot, we assumed that the average inflorescence density in the local portion of our transect was reflective of the average inflorescence density in the portion of the population serving as the seed source for our recruitment plots. We modeled recruitment using the number of seedlings as our response variable and including the average inflorescence density as a covariate. This allows the per-inflorescence recruitment rate to vary with the number of inflorescences. An alternative approach

would be to use the number of seedlings divided by the inflorescence density as our response variable, assuming that the per-inflorescence recruitment rate is constant. However, this latter approach greatly overestimated seedling recruitment under high inflorescence density, suggesting the existence of some limit on seedling recruitment with high seed production (e.g., safe-site limitation). For this reason, we used the first approach which implicitly includes safe-site limitation by allowing the per inflorescence recruitment rate to decrease with inflorescence density. With only three annual transitions per site, we were unable to test for lagged effects of inflorescence production on seedling recruitment.

Modeling growth with the beta distribution – We modeled growth as the distribution of sizes in the subsequent year as a function of size in the prior year. Although growth is commonly modeled with a normal distribution, size transitions in our data were not normally distributed even after log-transformation (Figure S1), and we were unable to even roughly meet the assumption of normality with other transformations. Instead, size transitions were positively skewed for small plants and negatively skewed for large plants, reflecting the fact that small plants cannot shrink below an absolute minimum size whereas large plants can often shrink much more dramatically than they can grow in a single year. We used two approaches to appropriately model these skewed size transitions:

First, we modeled the size distribution of surviving seedlings using the empirical probability density (quantified with the cumulative density and density functions and using default parameters), rather than assuming a particular distribution.

Second, we modeled the size transitions of all other individuals using an approach recently suggested by Peterson et al. (2019) based on the beta distribution. We transformed each

value of size at time t+1 (y_{t+1}) to a (0,1) interval determined by size-dependent minimum and maximum sizes as:

$$y'_{t+1} = (y_{t+1} - y_{min|\,y_t}) \,/\, (y_{max|\,y_t} - y_{min|\,y_t}) \qquad \text{Eq. S1}$$

where $y_{max|\,y_t}$ and $y_{min|\,y_t}$ are the maximum and minimum sizes at time t+1 conditional on starting size y_t. We estimated the 0.5 and 99.5 quantiles of size for each species as a linear function of starting size using quantile regression. In some cases, such as the growth of large plants, sizes were clustered very close to the maximum threshold, so we widened these thresholds slightly by subtracting or adding 0.5; these values were used as the size-dependent minimum and maximum values to transform size to a (0,1) interval. This approach left a small fraction of data points outside the (0,1) interval, so we adjusted any values of $y'_{t+1} \leq 0.01$ or ≥ 0.99 to 0.01 and 0.99, respectively. We fit beta regressions, using the betareg package in R v. 3.6.0, to model the mean and precision of the transformed sizes at time t+1 (y'_{t+1}) as functions of the starting size (y_t). The beta-distribution can take a wide range of shapes, from symmetric distributions to positively or negatively skewed distributions, depending on the mean and precision. We considered models with effects of starting size, site, and year on both the mean and precision, and used AIC to select the best-supported models. We then used the size-dependent estimates of the mean, precision, minimum, and maximum sizes from the best-supported models to back-transform to the original size scale to obtain the distribution of size at time t+1 as a function of starting size at time t. Specifically, we obtained the mean and variance on the original data scale as:

$$\mu_{\,y_{t+1}|\,y_t} = (\mu_{\,y'_{t+1}|\,y_t}\,(y_{max|\,y_t} - y_{min|\,y_t})) + y_{min|\,y_t} \qquad \text{Eq. S2}$$

$$\sigma^2_{y_{t+1}|\,y_t} = \sigma^2_{y'_{t+1}|\,y_t}\,(y_{max|\,y_t} - y_{min|\,y_t})^2 \qquad \text{Eq. S3}$$

where $\mu_{\,y_{t+1}|\,y_t}$ and $\sigma^2_{y_{t+1}|\,y_t}$ are the mean and variance of y_{t+1}, conditional on starting size y_t. Additional details of this approach are given by Peterson et al. (2019).

Climate variables – We downloaded monthly mean temperature and precipitation data for each site from PRISM, as well as the soil water holding capacity from 0-30 cm depth from SSURGO USDA (https://www.nrcs.usda.gov/wps/portal/nrcs/detail/soils/survey/office/ssr12/tr/?cid=nrcs142p2_010596). From these, we calculated the actual evapotranspiration (AET), potential evapotranspiration (PET), the climatic water deficit (PET – AET, or CWD), total precipitation, and mean temperature in each month, and then further averaged or summed monthly values into winter (November - February) or spring (March – June) aggregate values. We chose these time periods because they correspond to the timing of germination in the winter and subsequent growth and reproduction in the spring. We focused on climate variables related to temperature, precipitation, and drought stress because of their demonstrated effects on PNW prairie plant performance (Pfeifer-Meister et al., 2016; Reed et al., 2019). As an alternative to CWD or AET, we also used principal components analysis in each time period to capture variation in temperature and precipitation in either the winter or spring. We used the first principal component, explaining 66-88% of the variation, as an alternative metric of drought. Preliminary data exploration showed that these climate variables were more strongly correlated with performance than other potential measures of drought stress (e.g., SPEI).

Structured population models – We modeled the survival, growth, and reproduction of 2+ year-olds using size-dependent vital rate models, and the size-independent survival and growth of a separate seedling class. Our models can be expressed in kernel notation as:

$$n(z', t+1) = \int_L^U G(z', z)s(z)n(z, t)\, dz + c(z')s_{sdl}B(t) \qquad \text{Eq. S4}$$

$$B(t+1) = \int_L^U p(z)f(z)n(z, t)\, dz \qquad \text{Eq. S5}$$

where n(z,t) is the number of 2+ year-olds with size z at time t and B(t) is the number of 1 year-old seedlings at time t. G(z',z) is the growth function describing the distribution of size z' reached in one time step by an individual with starting size z, s(z) is the probability of survival of an individual with size z. c(z') is the distribution of size z' reached in one time step by a surviving seedling, s_{sdl} is the size-independent probability of a seedling surviving to reach 2 years old. p(z) is the probability of an individual with size z producing at least 1 inflorescence, and f(z) is the predicted number of recruiting seedlings produced by a reproductive individual with size z, which is determined by the size-dependent number of inflorescences produced by a reproductive individual. The kernels were discretized into 200 size classes using the midpoint rule and the observed upper (U) and lower (L) size bounds for each species. Upper and lower size bounds were set to the observed minimum and maximum sizes extended by a small constant (±0.001). Growth functions G(z',z) and c(z') were discretized by taking the difference of the cumulative density function (CDF) for the two size class boundaries (e.g., Dibner et al., 2019). Eviction was prevented by re-normalizing the growth probabilities to sum to 1 (Williams et al., 2012).

Estimation of lifespan – We used the discretized matrices to estimate the average lifespan of each species as the age at which a new seedling has less than 1% probability of still being alive, using the mean matrix across all sites and years. These estimates ranged from 11 years for *Achnatherum* to 20 and 26 years for *Danthonia* and *Festuca*, respectively.

Elasticity analysis – To investigate patterns of elasticity, we grouped the 200 size classes into 10 size categories of 20 classes each. We computed the elasticity of λ to the underlying vital rates by simultaneously perturbing vital rates for all individuals within each of 10 broader size classes as well as seedlings. For each of the 11 classes (seedlings plus 10 broad size classes), we

perturbed the probability of survival, probability of reproduction, number of inflorescences, and number of seedlings per inflorescence separately by multiplying by 0.95 or 1.05 and using the average change in λ to calculate the elasticity.

For beta-distributed growth transitions, the mean and variance depend on the minimum and maximum size boundaries. We calculated the elasticity of λ to mean growth and variance in growth separately by perturbing the minimum and maximum size boundaries. First, we added or subtracted a small constant (0.1) to shift boundaries up or down while preserving the difference between them. This results in small perturbations to the mean of the beta-distributed growth transitions while preserving the variance. We next perturbed the boundaries by a small constant to slightly increase or decrease the variance of the beta-distributed growth transitions while preserving the mean. To do this, we increased or decreased the minimum size by multiplying by a constant ($\Delta_{min|\,y_t}$), and then calculated the proportional change in the maximum size that would preserve the mean value as:

$$\Delta_{max|\,y_t} = 1 - [\, y_{min|\,y_t} (1- \mu_{\,y'_{t+1}|\,y_t}) (\Delta_{min|\,y_t} - 1) (\mu_{\,y'_{t+1}|\,y_t}\, y_{max|\,y_t})^{-1} \,] \qquad \text{Eq. S6}$$

We multiplied the minimum and maximum size boundaries by $\Delta_{min|\,y_t}$ and $\Delta_{max|\,y_t}$, respectively, to perturb the variance while keeping the mean value constant. We used 0.95 or 1.05 for $\Delta_{min|\,y_t}$ and used the average change in λ to calculate the elasticity.

Elasticities were calculated for each site and annual transition to obtain a distribution of estimates.

Population growth as a function of climate and latitude - We explored how population growth varies as a function of both climate and latitudinal variation. Specifically, we calculated λ across a range of latitudes and multivariate climate conditions. We sampled 2000 sets of correlated climate values from a multivariate normal distribution defined by the means and

covariances of the climate variables in the dataset. These multivariate climate conditions were used as drivers to obtain estimates of λ across the observed range of latitudes in the dataset. Some novel combinations of climate and latitude yielded unrealistically high predictions of fecundity (due largely to the log link function of the negative binomial), so we enforced a ceiling on predictions of mean inflorescence production and seedling recruitment such that these mean vital rates did not exceed the maximum value observed for each species to prevent extrapolation to unrealistic growth rates in these simulations. We also tested the contribution of specific vital rates to changes in λ with latitude or climate by varying each vital rate separately while holding all other vital rates constant at their mean value.

SUPPLEMENTAL TABLES

Table S1: Description of the study sites. We monitored three perennial bunchgrasses: *Achnatherum lemmonii*, *Danthonia californica*, and *Festuca roemeri* from their approximate latitudinal range centers toward their northern range limits. *Danthonia* and *Festuca* reach their northern range limits at approximately 50.1° and 49.9° N, respectively. *Achnatherum* is exceedingly rare north of ~45.5° N, the northern end of the Willamette Valley in Oregon.

Species	Site	Latitude	Longitude	Elevation (m.a.s.l)	Description
Achnatherum	Agate Desert	42.42764	-122.888	385	Open prairie. Controlled burn in 2018.
Achnatherum	Cooper	45.44663	-122.876	161	Open prairie. Controlled burn in 2017.
Achnatherum, Danthonia, Festuca	French Flat	42.0949	-123.64	451	Open prairie on serpentine soil. Many nematode galls on *Danthonia*.
Achnatherum, Danthonia, Festuca	Hazel Dell	44.02518	-123.216	167	Open prairie.
Danthonia, Festuca	Horse Rock Ridge	44.29804	-122.878	730	Prairie on steep, rocky slopes surrounded by mixed woodland.
Festuca	Roxy Ann Peak	42.34913	-122.788	874	Prairie in small clearings in mixed woodland.
Danthonia, Festuca	Table Mountain	42.46721	-122.946	385	Open prairie. High invasive species cover. Controlled burn of *Danthonia* site in 2018.
Danthonia, Festuca	Upper Weir	46.90978	-122.708	159	Open prairie. High invasive species cover.
Achnatherum, Danthonia	Whetstone Prairie	42.40959	-122.909	383	Vernal pool prairie. High invasive species cover. Controlled burn of *Danthonia* site in 2018.
Festuca	Whidbey Island	48.20977	-122.624	66	Open prairie. Controlled burn in 2018.

Table S2: Best-supported categorical and climate-based vital rate models for three perennial bunchgrasses. For each species and vital rate, the best-supported models are shown from a series of categorical models using site and year as fixed effects, and a series of mixed models using climate variables and latitude, and treating site as a random effect. N gives the sample size for each species and vital rate used in model-fitting. Pseudo-R^2 values are given for each model, using Nagelkerke's R^2 for categorical models (glms) and the marginal (R^2_m, fixed effects only) and conditional R^2 (R^2_c, fixed and random [i.e., site] effects) for mixed models (glmms) (Nakagawa and Schielzeth 2013). R^2 values for growth models are the correlation between the linear predictor and the link-transformed response (Cribari-Neto and Zeileis 2010). Note: climate-based growth models always include site as a fixed effect, rather than a random effect, for both the mean (μ) and precision (ϕ). Recruitment models always include inflorescence density as a predictor. Categorical seedling survival models only considered site for *Achnatherum* and *Danthonia* and a single species-wide estimate for *Festuca* due to sample size constraints, limiting their explanatory power relative to climate-based models. * indicates an interaction between two variables, such that the main effects are also included in the model. Parentheses indicate interactions with all variables inside the parentheses.

**Vital rate**	**N**	**Categorical model**	**R^2**	**Climate model**	**R^2_m**	**R^2_c**
Achnatherum lemmonii						
Survival of established plants	3086	Size*Site*Year	.511	Size*($PC1_{Spring}$ + $PC1_{Spring}^2$) + Lat*$PC1_{Spring}$	.326	.371
Growth	2408	μ: Size*Site*Year, ϕ: Site + Year	.408	μ: Size + CWD_{Spring} + CWD_{Spring}^2, ϕ: Size + CWD_{Spring} + CWD_{Spring}^2		.356
Reproduction	4357	Size*Site*Year + $Size^2$*Year	.741	CWD_{Spring}*(Size + $Size^2$)	.688	.694

Inflorescences	2368	Site*(Size + $Size^2$*Year)	.890	PC_{Spring}*(Size + Lat) + $Size^2$ + Lat*PC_{Spring}^2	.404	.644
Recruitment	320	Site*Year	.873	T_{Winter}	.087	.644
Seedling survival	274	Site	.244	T_{Winter}	.156	.441
Danthonia californica						
Survival of established plants	3007	Size*Site*Year	.396	CWD_{Spring}*(Size + Lat)	.177	.239
Growth	2575	μ: Size*Site*Year, ϕ: Size + Site + Year	.309	μ: Size*CWD_{Spring} + CWD_{Spring}^2, ϕ: Size + CWD_{Spring}		.261
Reproduction	4021	Size*Site*Year	.584	PC_{Spring}*($Size^2$ + Lat) + PC_{Spring}^2*(Size + Lat)	.424	.511
Inflorescences	2239	Site*(Size + Year) + $Size^2$*Year	.890	Size + $Size^2$*(P_{Spring} + P_{Spring}^2)	.457	.527
Recruitment	332	Site*Year	.485	P_{Spring}	.133	.194
Seedling survival	330	Site	.186	P_{Spring}	.080	.237
Festuca roemeri						
Survival of established plants	3985	Size*Site*Year	.423	CWD_{Spring}*(Size + Lat)	.258	.264
Growth	3563	μ: Size*Site*Year, ϕ: Size + Site + Year	.507	μ: Size*(T_{Spring} + T_{Spring}^2), ϕ: Size + T_{Spring}		.459
Reproduction	5494	Size*Site + $Size^2$*Site + $Size^2$*Year + Site*Year	.512	Size + $Size^2$ + Lat*($PC1_{Spring}$ + $PC1_{Spring}^2$)	.417	.453
Inflorescences	2264	Size*Site*Year + $Size^2$	.732	Lat*CWD_{Spring} + $Size^2$	.376	.522
Recruitment	530	Site*Year	.562	T_{Spring}	.318	.472
Seedling survival	96	Intercept-only		AET_{Spring}	.138	.138

Table S3: ANCOVA table for patterns of population growth for three perennial bunchgrasses relative to latitude. Type 3 sums of squares (SS), degrees of freedom (Df), and corresponding F-tests are shown for the asymptotic population growth rate for each annual transition (λ) as well as the transient stochastic population growth rate (λ_s).

	λ				λ_s			
	Df	SS	F	P	Df	SS	F	P
Latitude	1	0.183	10.23	0.002	1	0.092	10.12	0.008
Species	2	0.098	2.75	0.074	2	0.056	3.08	0.083
Latitude * Species	2	0.102	2.85	0.068	2	0.057	3.10	0.082
Error	48	0.857			12	0.110		

Table S4: Coefficients and standard errors from the best-supported climate-based vital rate models for three perennial bunchgrasses (see Table 1 for best-supported model structure). Adult and seedling survival and the probability of reproduction are modeled with a binomial distribution and logit link function. The number of inflorescences and the number of recruited seedlings are modeled with a negative binomial distribution and log link function. The mean (μ) and precision (ϕ) in growth are modeled with a beta distribution and a log-log link for the mean and a log link for the precision (see Appendix S1 for details). Seedling recruitment models always include inflorescence density as a covariate; this coefficient is given in the size column. Growth models include site as a fixed effect, and these values are given in Table S5.

**Vital rate**	Intercept	Size (Infl)	Size^2	Clim	Clim^2	Lat	Size* Clim	Size* Clim^2	Size^2* Clim	Size^2* Clim^2	Lat* Clim	Lat* Clim^2
Achnatherum lemmonii												
Survival of established plants	-1.690 (0.346)	0.830 (0.047)		-0.066 (0.153)	0.315 (0.088)	-0.908 (0.296)	-0.124 (0.026)	-0.046 (0.023)			-0.864 (0.138)	0.830 (0.047)
Growth μ	-0.502 (0.047)	0.230 (0.007)		-0.135 (0.033)	0.227 (0.019)							
Growth ϕ	1.207 (0.105)	0.075 (0.015)		-0.251 (0.070)	0.195 (0.038)							
Reproduction	-5.056 (0.328)	1.821 (0.152)	-0.088 (0.019)	1.401 (0.352)			-0.647 (0.173)		0.057 (0.021)			
Inflorescences	3.375 (7.016)	0.094 (0.083)	0.043 (0.008)	5.443 (1.252)	2.104 (0.929)	-0.079 (0.162)	0.031 (0.011)				-0.136 (0.029)	-0.051 (0.022)
Recruitment	-0.396 (0.580)	0.086 (0.032)		-0.669 (0.159)								
Seedling survival	-2.903 (0.955)			-2.124 (0.600)								
Danthonia californica												

Survival of established plants	0.510 (0.373)	0.560 (0.033)		-1.253 (0.208)		-1.155 (0.323)	0.138 (0.033)				-0.616 (0.176)	
Growth μ	0.102 (0.042)	0.187 (0.007)		-0.191 (0.041)	0.080 (0.018)		0.047 (0.008)					
Growth ϕ	1.551 (0.092)	0.218 (0.016)		-0.225 (0.078)								
Reproduction	-1.763 (0.380)	0.727 (0.083)	0.025 (0.015)	0.408 (0.117)	-0.084 (0.053)	0.435 (0.322)		0.065 (0.018)	0.029 (0.006)		0.745 (0.096)	0.522 (0.062)
Inflorescences	-0.078 (0.159)	0.260 (0.058)	0.026 (0.009)	0.110 (0.065)	-0.020 (0.025)				-0.022 (0.003)	0.009 (0.002)		
Recruitment	-0.064 (0.176)	0.079 (0.026)		-0.360 (0.157)								
Seedling survival	-1.273 (0.488)			0.931 (0.364)								
Festuca roemeri												
Survival of established plants	-0.826 (0.219)	0.747 (0.041)		-1.361 (0.218)		-1.120 (0.193)	0.247 (0.044)				-0.652 (0.183)	
Growth μ	-0.621 (0.039)	0.292 (0.006)		-0.411 (0.037)	0.236 (0.024)		0.058 (0.006)	-0.041 (0.004)				
Growth ϕ	1.928 (0.094)	0.111 (0.012)		-0.187 (0.045)								
Reproduction	-5.583 (0.368)	1.206 (0.115)	-0.045 (0.011)	-0.542 (0.072)	0.083 (0.025)	0.032 (0.202)					-0.194 (0.061)	-0.127 (0.059)
Inflorescences	-0.417 (0.357)	0.033 (0.104)	0.035 (0.008)	-0.515 (0.050)		-0.275 (0.167)					-0.370 (0.056)	
Recruitment	-1.392 (0.311)	0.072 (0.022)		-1.098 (0.217)								
Seedling survival	-0.802 (0.258)			1.197 (0.344)								

Table S5: Coefficients and standard errors for fixed effects of site from the best-supported climate-based growth models for three perennial bunchgrasses (see Table 1 for best-supported model structure). Coefficients are given for the mean (μ) and precision (ϕ) of beta-distributed growth (see Appendix S1 for details). The reference level for each species is given in bold; these are equivalent to the intercepts reported in Table S4.

	Achnatherum lemmonii		*Danthonia californica*		*Festuca roemeri*	
	μ	ϕ	μ	ϕ	μ	ϕ
Agate Desert	**-0.502 (0.047)**	**1.207 (0.105)**				
Cooper	-0.482 (0.095)	-0.753 (0.186)				
French Flat	0.164 (0.037)	0.613 (0.082)	**0.102 (0.042)**	**1.551 (0.092)**	**-0.621 (0.039)**	**1.928 (0.094)**
Hazel Dell	-0.255 (0.076)	-0.481 (0.154)	0.131 (0.053)	0.053 (0.128)	0.045 (0.030)	-0.091 (0.078)
Horse Rock			-0.384 (0.089)	-0.897 (0.200)	-0.549 (0.057)	-1.333 (0.109)
Roxy Ann					-0.049 (0.029)	0.107 (0.083)
Table Mountain			0.090 (0.035)	0.069 (0.084)	-0.033 (0.045)	-0.041 (0.101)
Upper Weir			-0.312 (0.073)	-0.820 (0.164)	-0.328 (0.045)	-0.956 (0.100)
Whetstone Prairie	0.349 (0.041)	0.324 (0.082)	0.099 (0.040)	-0.402 (0.087)		
Whidbey Island					-0.160 (0.043)	-0.907 (0.093)

Table S6: Correlations in population growth rate estimates from categorical vs. climate-based vital rate models. Climate-based population growth rates are from the best-supported models allowing interactions between latitude and climate as well as a random site effect (left), the best-supported models that exclude latitude (center), and the best-supported models that exclude latitude or site effects (right). Values are the Pearson correlation coefficients for λ estimates produced by these climate-based models vs. those based on categorical vital rate models that treat site and year as fixed effects to most flexibly model spatiotemporal variation in demography. Note: all correlations are significantly different from zero ($P < .05$).

	Latitude* Climate + Site	Climate + Site	Climate
Achnatherum	.946	.913	.774
Danthonia	.833	.798	.653
Festuca	.829	.766	.500

SUPPLEMENTAL FIGURES

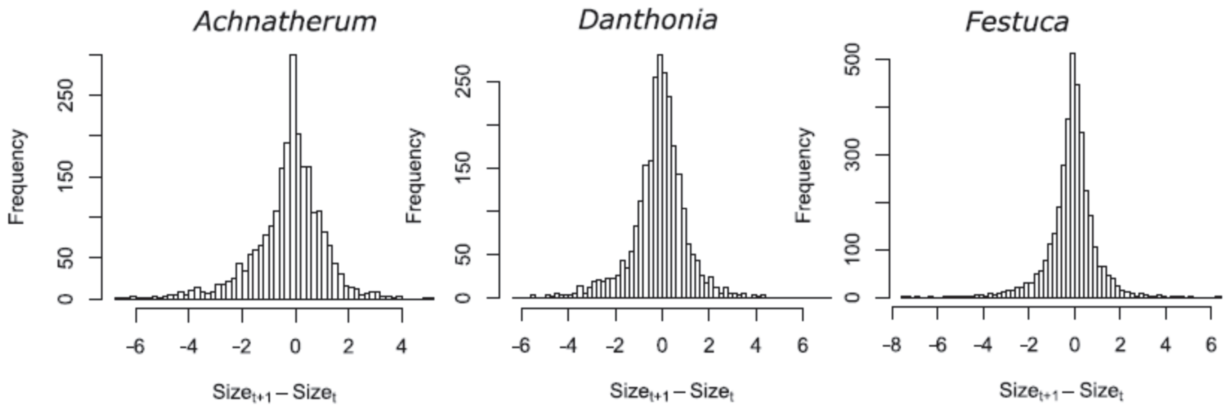

Figure S1: Size transitions are negatively skewed for three perennial bunchgrasses. Values are the difference in log(area) for an individual over one year.

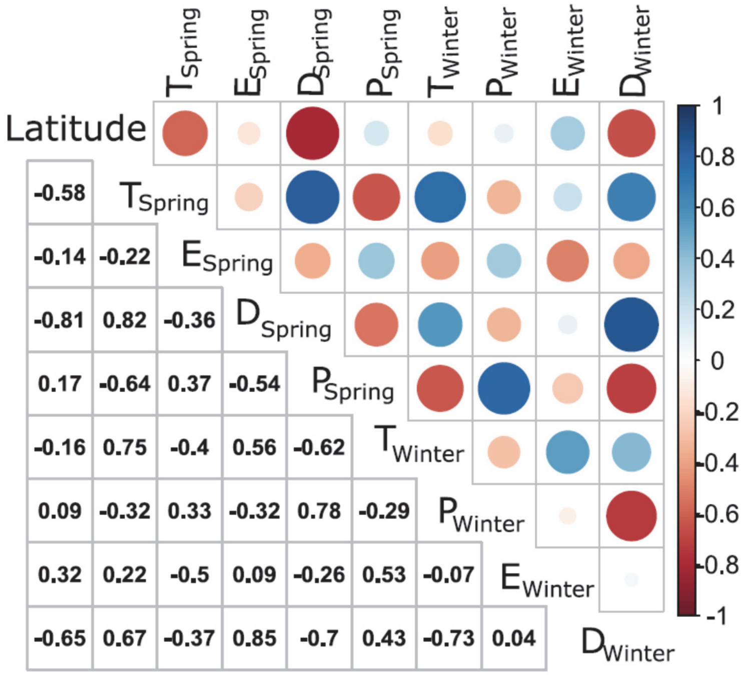

Figure S2: Correlations among latitude and climate variables. Spring temperature and climatic water deficit are significantly negatively correlated with latitude. Climate variables are temperature (T), actual evapotranspiration (E), climatic water deficit (D), and precipitation (P) in winter (November – February) and spring (March – June). Correlations are calculated across all demographic study sites (N=10, see Figure 1 map) and years (2015-2018).

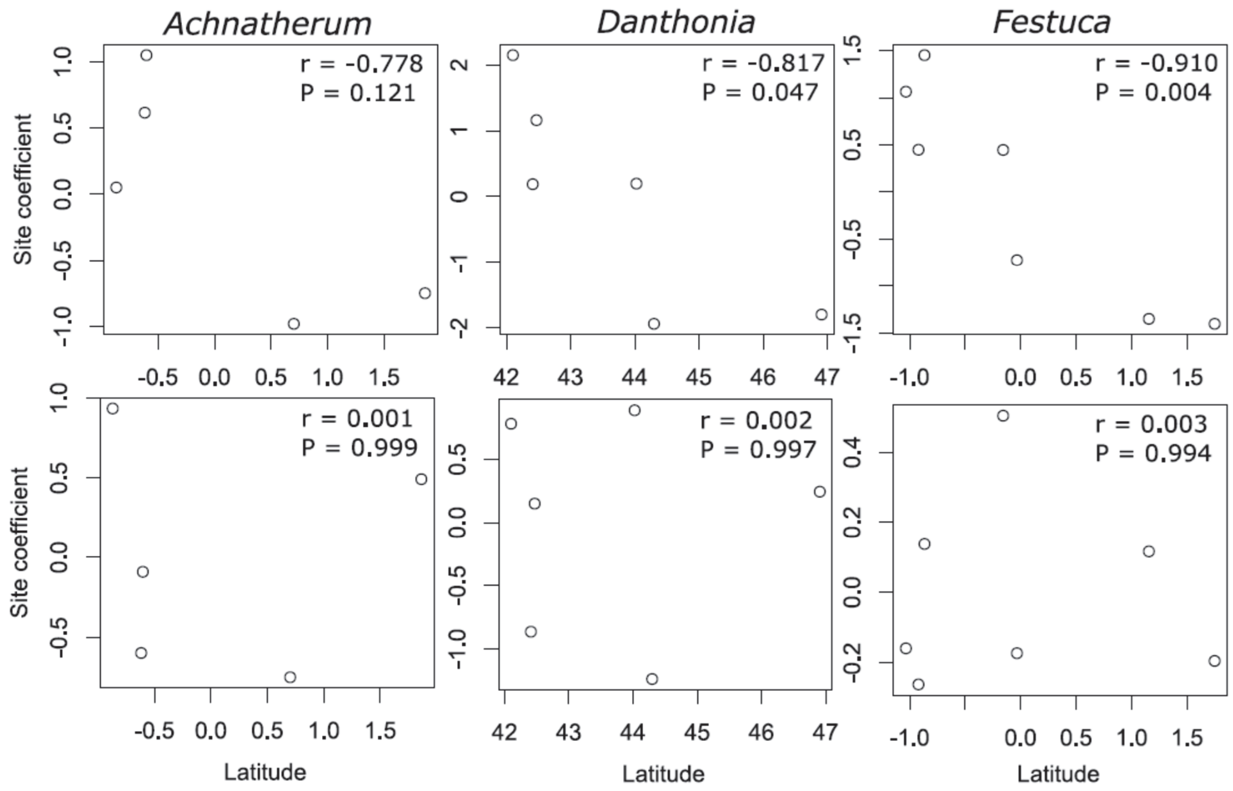

Figure S3: Random site coefficients are strongly correlated with latitude even after accounting for climate effects. Coefficients are from models of post-seedling survival for each species that include climate effects and random site effects but without (top panels) or with (bottom panels) a fixed effect of latitude. Correlations between site coefficients and latitude in the top panels indicate latitudinal variation in performance not captured by climate variables; these correlations are removed by including latitude as a fixed effect in the models (bottom panels).

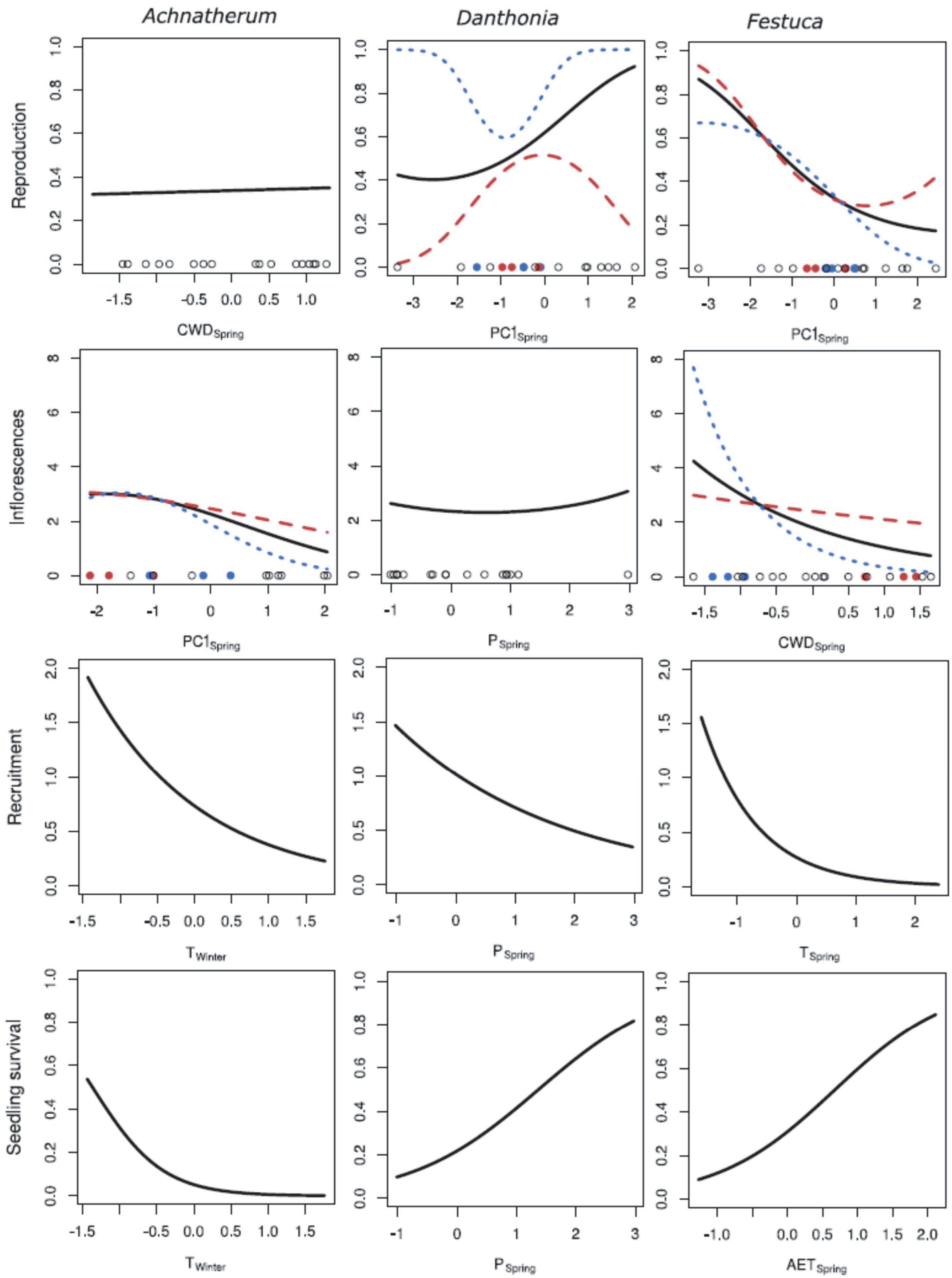

Achnatherum
Danthonia
Festuca
Reproduction
Inflorescences
Recruitment
Seedling survival
CWD_Spring
PC1_Spring
PC1_Spring
PC1_Spring
P_Spring
CWD_Spring
T_Winter
P_Spring
T_Spring
T_Winter
P_Spring
AET_Spring

Figure S4: Additional fitted vital rate responses to climate drivers across three perennial bunchgrasses (others shown in Figure 3 in the main text). Lines give the predicted vital rate responses from the best-supported climate models (Table 2), and points show the distribution of climate values observed across sites and years, for Left) *Achnatherum lemmonii*, Center) *Danthonia californica*, and Right) *Festuca roemeri*. Vital rates are shown for average-sized individuals and the mean latitude across all study sites for a given species (solid lines). Where there were significant latitude by climate interactions, we also show vital rates for the minimum (red dashed) and maximum (blue dotted) latitudes across study sites for each species, and highlight the observed climate values for these sites (red = minimum, blue = maximum). From top to bottom, vital rates are the 1) probability of reproduction, 2) number of inflorescences if reproductive, 3) number of seedlings per inflorescence, and 4) seedling survival.

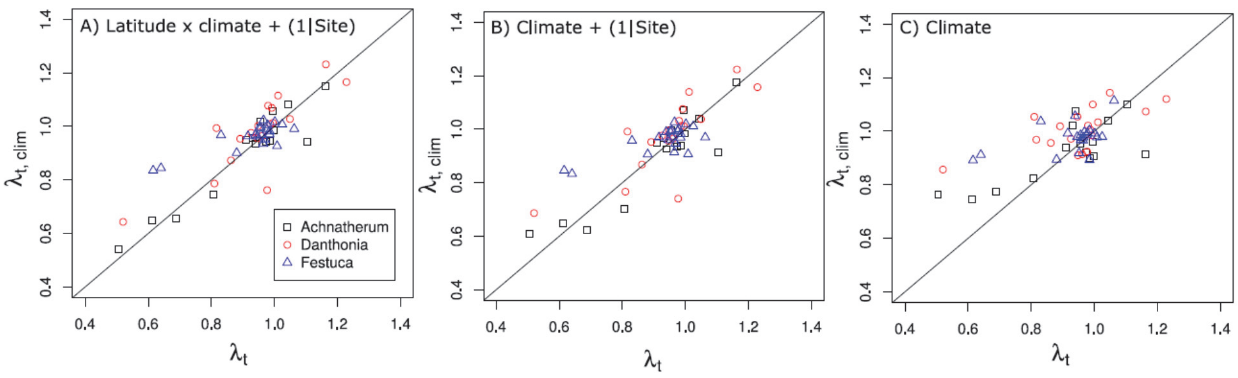

Figure S5: Correlation between population growth rates from climate-based vital rate models and from categorical models. Climate-based population growth rates are from the best-supported models allowing interactions between latitude and climate as well as a random site effect (A), the best-supported models that exclude latitude (B), and the best-supported models that exclude latitude or site effects (C). Correlation coefficients are given in Table 3.

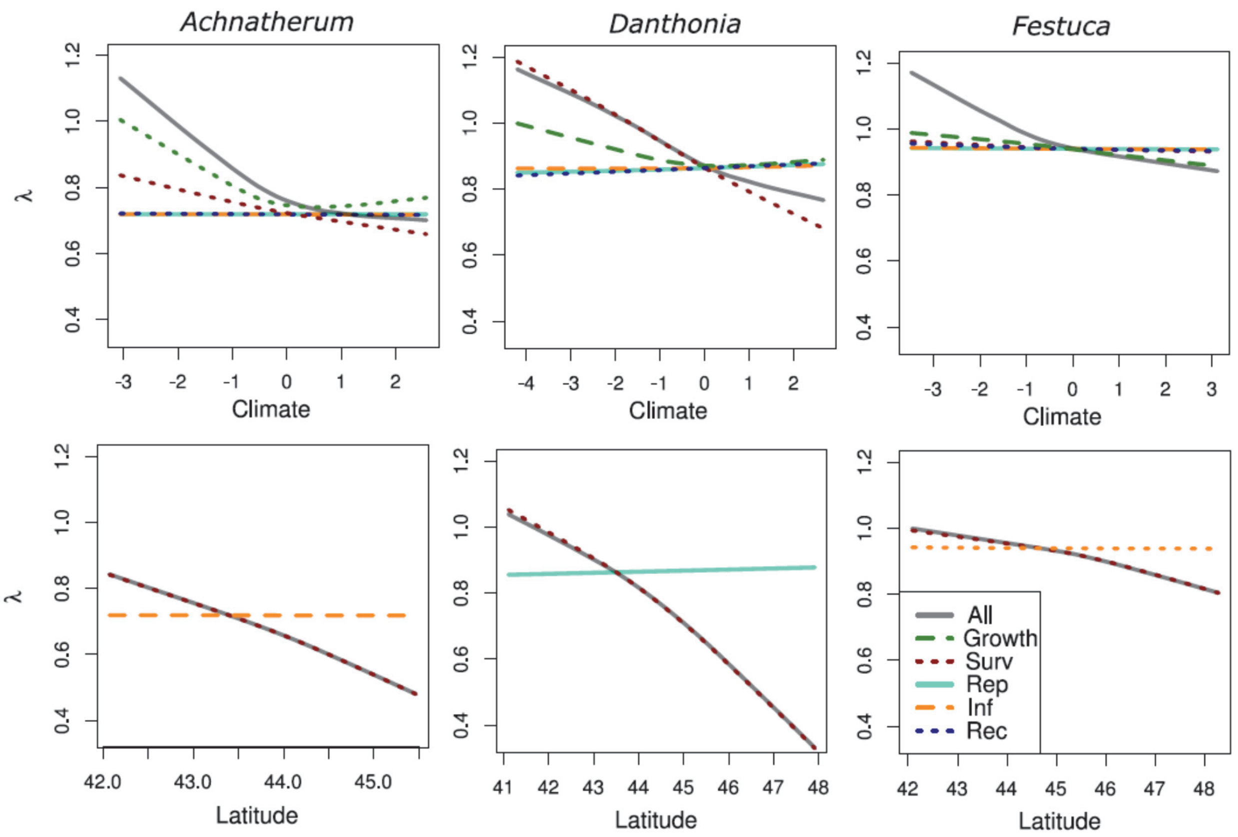

Figure S6: Sensitivity of population growth rate (λ) to climate and latitude drivers for three perennial bunchgrasses. Lines show loess fits of the relationship between λ and either 2000 correlated multivariate climate conditions (top), or latitude (bottom) when varying vital rates individually or all vital rates together. Only vital rates that are influenced by either climate or latitude in the best-supported climate models (see Table 1) were varied individually. Multivariate climate conditions are shown as a principal component of all climate variables which captures variation from cooler/wetter conditions (negative values) to warmer/drier conditions (positive values). Lines show responses when varying all vital rates (All: grey solid), mean and variance in growth (Growth: green dashed), survival of established individuals and seedlings (Surv: red dotted), probability of reproduction (Rep: teal solid), number of inflorescences (Inf: orange dashed), or the number of recruited seedlings for a given inflorescence density (Rec: blue dotted).

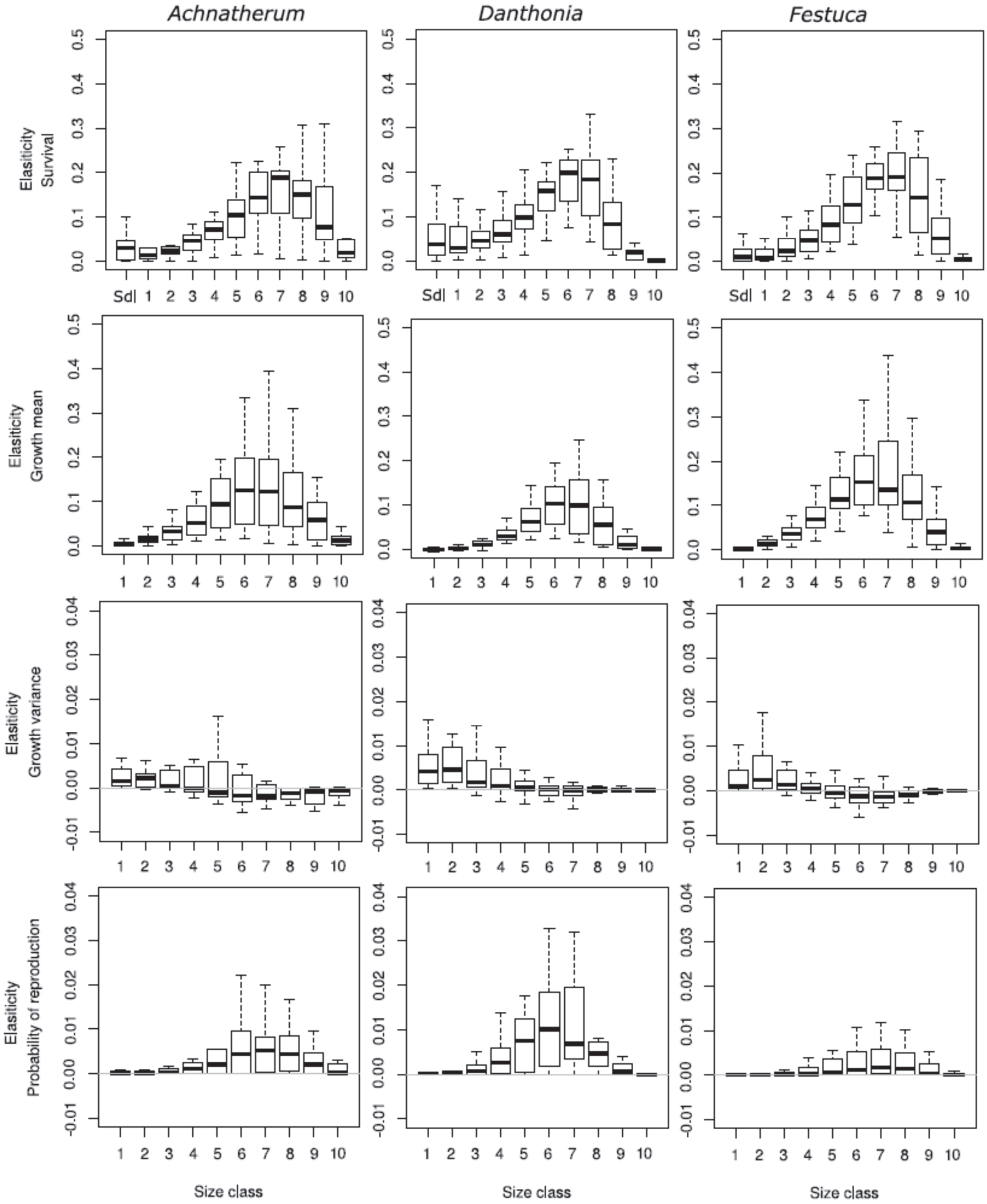

Achnatherum
Danthonia
Festuca
Elasticity
Survival
Elasticity
Growth mean
Elasticity
Growth variance
Elasticity
Probability of reproduction
Sdl 1 2 3 4 5 6 7 8 9 10
Size class

Figure S7: Elasticity of demographic parameters for three perennial bunchgrasses. Elasticity values were estimated by perturbing values within 10 evenly divided size classes for each annual matrix in each site. Note that the elasticity of the number of inflorescences is equivalent to that for the probability of reproduction, and that the elasticity of the number of seedlings per inflorescence is equivalent to that for seedling survival. Box plot components are mid line, median; box edges, upper and lower quartiles; whiskers, extending to the upper and lower fences at 1.5 times the interquartile range.

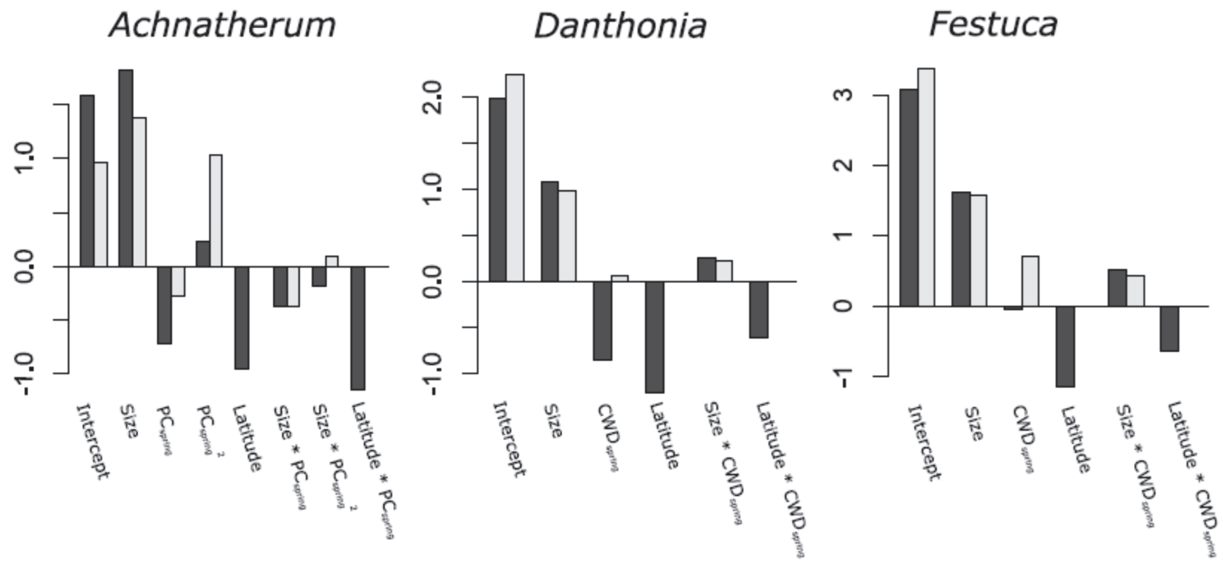

Figure S8: Comparison of standardized coefficients with and without considering latitude and site effects. Black bars show the coefficients from the best-supported climate model of survival for each species, including latitude as a fixed effect and site as a random effect. Grey bars show the coefficients from a model with the same climate and size variables but without latitude or a random site effect. The magnitude and direction of inferred climate effects is different when focusing on climate alone and ignoring latitude or site effects not captured by climate.

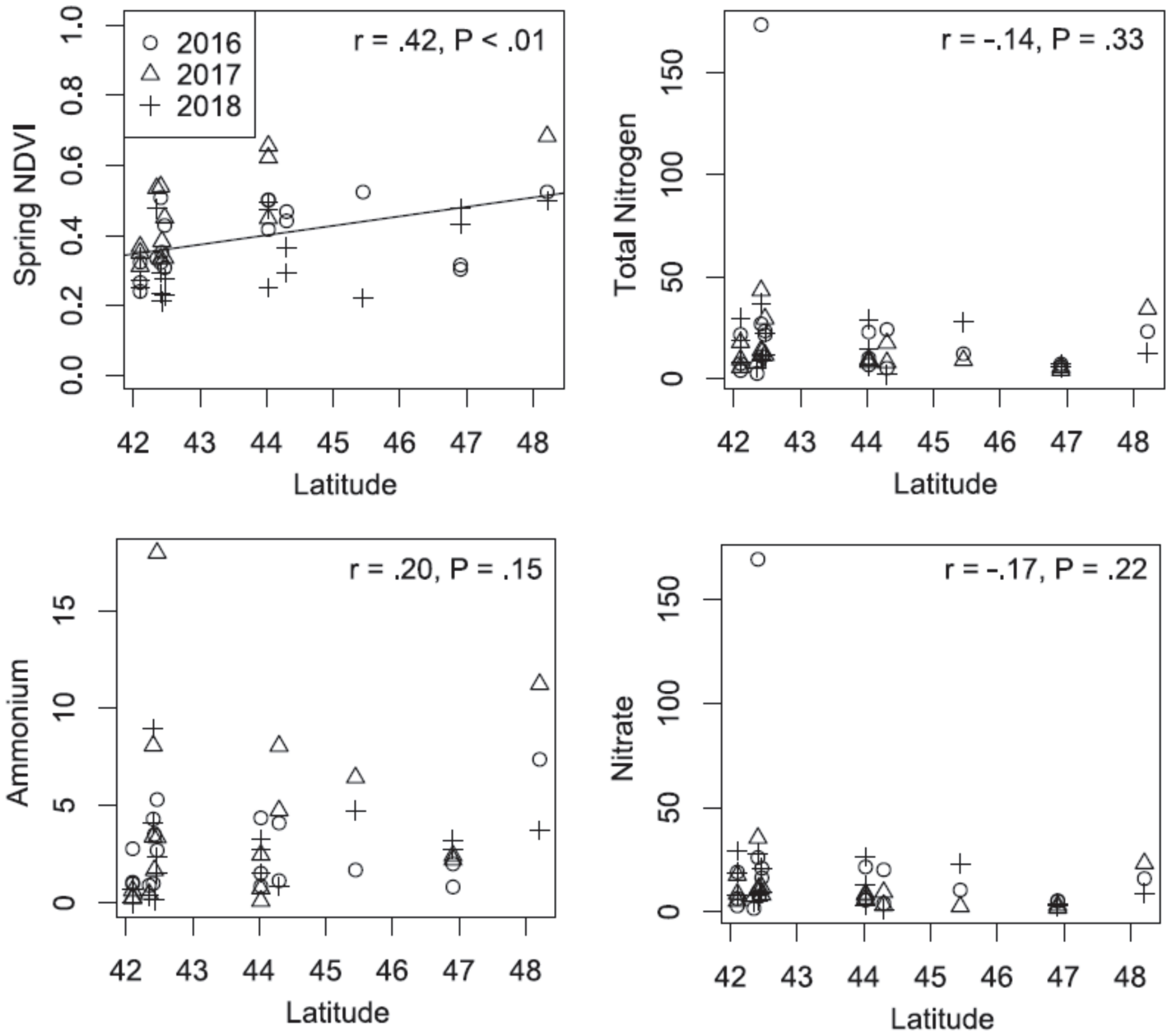

Figure S9: Relationships of other environmental drivers with latitude. We measured NDVI along the demographic transects at each study site in the spring of each year using a handheld Crop Circle ACS-430 sensor (Holland Scientific Inc.) and took the average value across 10 measurements for each site and year. Note that NDVI was not measured in 2015 or for several sites in 2017. We measured soil ammonium, nitrate, and total inorganic nitrogen concentration at each study site in the spring (March – July) of each year by burying resin strip probes that absorb soil cations and anions (PRS Probes, Western Ag, Canada). Lines indicate statistically significant relationships.

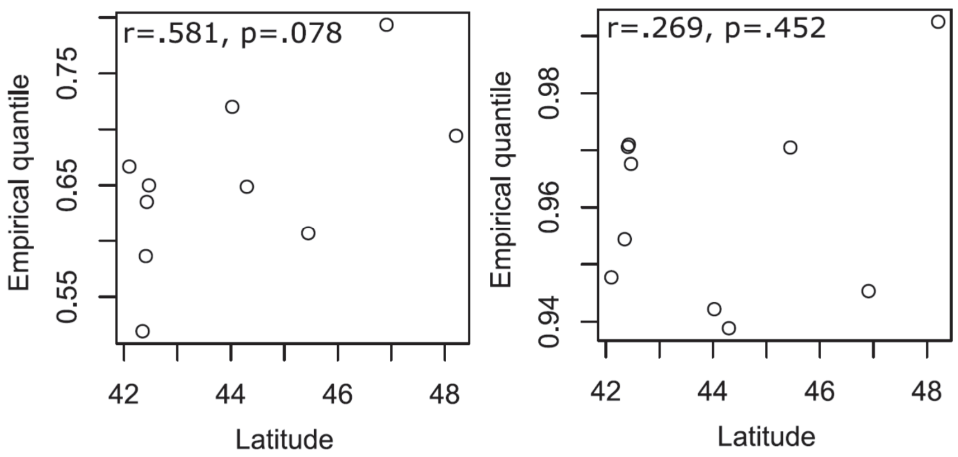

Figure S10: Empirical quantiles of extreme climate conditions during the study period relative to the distribution of historical climate conditions at each site. Values for the driest (CWD_{Spring}, left) or warmest (T_{Spring}, right) years at each site during the study period (2015-2018) as quantiles of the empirical distribution of historical values at each site from 1895 – 1995. There is a trend that the driest years were more extreme relative to historical climate conditions at higher latitudes.

References

Cribari-Neto, F. and A. Zeileis. 2010. Beta Regression in R. Journal of Statistical Software 34:1–24.

Dibner, R.R., M.L. Peterson, A. Louthan, and D. Doak. 2019. Multiple mechanisms confer stability to isolation populations of a rare endemic plant. Ecological Monographs 89:e01360.

Nakagawa, S. and H. Schielzeth. 2013. A general and simple method for obtaining R^2 from generalized linear mixed-effects models. Methods in Ecology and Evolution 4:133–142.

Peterson, M.L., W. Morris, C. Linares, and D. Doak. 2019. Improving structured population models with more realistic representations of non-normal growth. Methods in Ecology and Evolution 10:1431–1444.

Pfeifer-Meister, L., S.D. Bridgham, L.L. Reynolds, M.E. Goklany, H.E. Wilson, C.J. Little, A. Ferguson, and B.R. Johnson. 2016. Climate change alters plant biogeography in Mediterranean prairies along the West Coast, USA. Global Change Biology 22:845–855.

Reed, P.B., L.E. Pfeifer-Meister, B.A. Roy, B.R. Johnson, G.T. Bailes, A.A. Nelson, M.C. Boulay, S.T. Hamman, and S.D. Bridgham. 2019. Prairie plant phenology driven more by temperature than moisture in climate manipulations across a latitudinal gradient in the Pacific Northwest, USA. Ecology and Evolution 9:3637–3650.

Williams, J.L., T.E. Miller, and S.P. Ellner. 2012. Avoiding unintentional eviction from integral projection models. Ecology 93:2008–2014.
